# Residual Asymmetry Modeling and Joint Time–Frequency Estimation for High-Dynamic Two-Way Microwave Links

**DOI:** 10.3390/s26113470

**Published:** 2026-05-31

**Authors:** Zhijuan Hao, Huabing Wu

**Affiliations:** 1 National Time Service Center, Chinese Academy of Sciences, Xi’an 710600, China; haozhijuan@ntsc.ac.cn; 2 University of Chinese Academy of Sciences, Beijing 100049, China; 3 Key Laboratory of Time Reference and Applications, Chinese Academy of Sciences, Xi’an 710600, China

**Keywords:** high dynamics, microwave two-way time synchronization, joint time–frequency observation, residual asymmetric error, link-state-information-assisted IMM-IEKF

## Abstract

High-precision time synchronization among high-dynamic platforms is an important foundation for distributed detection, cooperative sensing, and networked operation of high-speed mobile platforms. In high-dynamic two-way microwave links, rapid variations in propagation geometry, Doppler-related frequency offsets, and link-quality fluctuations can break the approximate symmetry between uplink and downlink propagation. Although geometric and motion compensation can remove the dominant propagation-asymmetry term, residual asymmetric errors caused by propagation modeling errors, compensation mismatch, and link degradation may still remain and couple into clock-offset estimation, thereby reducing synchronization stability and accuracy. To address this problem, this paper proposes a modeling and joint estimation method for residual asymmetric errors in high-dynamic two-way microwave links. The post-compensation residual error is modeled as a recursively estimable dynamic state, and its rate of change is introduced to characterize the short-term evolution of the residual term. Meanwhile, a four-timestamp and frequency-offset joint observation model is constructed, in which frequency-offset information is used as an observation-level auxiliary constraint to enhance local separability among the clock offset, frequency offset, and residual link state. On this basis, a link-state-information-assisted IMM-IEKF is adopted to realize online joint estimation of clock parameters and link residual errors. Under the equivalent stochastic-error simulation setting, the proposed method effectively suppresses post-compensation residual asymmetric errors and achieves sub-nanosecond synchronization accuracy under strong-dynamic and degraded-link conditions.

## 1. Introduction

In conventional microwave two-way time synchronization, the uplink and downlink propagation paths are usually assumed to be approximately symmetric, and the link state is considered relatively stable within a short time interval [1]. Based on this assumption, a four-timestamp exchange can cancel the dominant common propagation delay and obtain the clock-offset estimate between nodes. However, in high-dynamic scenarios, the relative motion of platforms continuously changes the signal propagation geometry. The platform positions corresponding to signal transmission, forwarding, and reception are different, and the uplink and downlink signals no longer propagate along fully reciprocal paths. Meanwhile, Doppler-related frequency offsets, attitude disturbances, receiver tracking errors, and link-noise fluctuations further amplify the propagation difference between the two directions, causing four-timestamp observations to contain clock-related, motion-related, and link-related terms simultaneously [2,3,4].

Existing studies usually reduce the dominant propagation-asymmetry term through geometric and motion compensation. However, residual asymmetric errors may still remain in the compensated link. These residual errors mainly arise from propagation modeling errors, trajectory and velocity information errors, local compensation mismatch, receiver tracking errors, and link-quality degradation. Since these factors are closely related to platform motion, propagation geometry, and link state, the compensated residual asymmetric error is not suitable to be simply treated as white noise or a fixed bias. During strong maneuvering phases, rapid changes in platform velocity and acceleration alter the non-reciprocity between uplink and downlink propagation, leading to dynamic drift in the residual error. During link-degradation phases, increased observation noise, degraded frequency-offset measurement quality, local missing observations, and abnormal innovations further make the residual term non-stationary. Therefore, incorporating the compensated residual completely into observation noise would ignore its trackable dynamic structure, while treating it as a fixed bias would make it difficult to adapt to error evolution under strong-dynamic and degraded-link conditions [5].

From the perspective of observation structure, the four-timestamp observation after geometric and motion compensation contains not only the clock-offset term but also the post-compensation residual asymmetric error and observation noise. The clock-offset term and the residual link term enter the same observation in an additive form. When only compensated four-timestamp observations are used, these two terms lack sufficient independent constraints and are prone to mixing, leading to insufficient local separability. Strong maneuvering and link degradation further increase the time variability and uncertainty of the residual link term, making the implicit assumption that the compensated residual can be approximated as noise or an unreliable fixed bias [3,4,6]. Therefore, the key problem in high-dynamic microwave two-way time synchronization is no longer limited to geometric compensation of the dominant propagation-asymmetry term. It also involves how to impose observation constraints, conduct dynamic modeling, and perform online estimation for the residual asymmetric error that remains after compensation and is coupled with the clock offset.

For microwave two-way time synchronization, existing studies have extended conventional symmetric two-way transfer models toward dynamic motion compensation and refined link-error correction. In terms of propagation compensation, Tseng et al. used satellite ephemerides to correct the Sagnac effect and diurnal variation model, thereby addressing non-reciprocal path errors caused by satellite motion [3]. Sun et al. established a dynamic two-way transfer model including platform trajectory and propagation-delay variation, providing a basis for two-way time transfer between moving platforms [7]. Zhang et al. pointed out that the Doppler effect not only affects propagation frequency but may also introduce bias through the receiver code-measurement process, indicating that high-dynamic link errors are also related to receiver tracking, signal processing, and measurement mechanisms [4]. Fujieda et al. and Jiang et al. further improved two-way link stability through carrier-phase TWSTFT, software-defined radio receivers, redundant link processing, and indirect link combinations [8,9]. These studies have promoted the development of dynamic time synchronization from simple propagation-delay cancellation toward the integrated correction of motion-induced propagation effects, receiver biases, and link stability.

Meanwhile, frequency synchronization, phase synchronization, and propagation-aware correction have also been gradually introduced into dynamic time synchronization. Studies on distributed antenna arrays, cooperative radar, and GPS-denied networks have shown that frequency offset and phase information can reflect relative motion, propagation variations [10], and local oscillator frequency differences, thereby providing additional constraints for time synchronization under dynamic conditions [11]. Doppler-assisted time synchronization studies have also shown that frequency-related observations can supplement the information deficiency of four-timestamp observations from the dimension of temporal variation rates [4,12]. Therefore, frequency-offset information should not be used only for frequency estimation or state prediction. It can also be introduced as an observation-level auxiliary constraint into the joint estimation of clock offset, frequency offset, and residual link state so as to alleviate the coupling between the clock-offset term and the residual link term.

Although existing studies have made progress in dominant propagation-error compensation, auxiliary observation introduction, and recursive clock-parameter estimation, they usually lack explicit state representation and independent observation constraints for the link asymmetric errors that remain after geometric and motion compensation. Existing methods often incorporate the compensated residuals into observation noise, empirical biases, or unmodeled disturbances [13]. This treatment may be acceptable under low-dynamic or link-stable conditions, but it is difficult to adapt to the continuous evolution of residual errors under strong maneuvering and link degradation. Without explicit state modeling, the filter has difficulty distinguishing whether the error comes from clock-state variations or link-residual variations. Without additional observation constraints, the clock-offset term and the residual link term remain prone to mixing in single-link four-timestamp observations. Therefore, dynamic modeling of post-compensation residual asymmetric errors and enhancement of their separability from clock parameters remain key issues in high-dynamic microwave two-way time synchronization.

Frequency-offset observations provide a new source of constraints for this problem. Frequency-offset information not only reflects clock-frequency deviations but also contains frequency-response information caused by relative motion and propagation variation. Compared with a single four-timestamp observation, frequency-related observations can provide supplementary constraints from the temporal variation-rate dimension. Existing studies on Doppler-assisted time synchronization and distributed array synchronization have shown that frequency-related observations help improve time synchronization performance and cooperative-system consistency under dynamic conditions [13,14].

Based on the above analysis, this paper focuses on the modeling and estimation of post-compensation residual asymmetric errors in high-dynamic two-way microwave links, and it constructs a processing framework that combines joint time–frequency observation, residual-state modeling, and link-state-information-assisted estimation. Specifically, a four-timestamp and frequency-offset joint observation model is constructed, in which compensated four-timestamp observations and frequency-offset-assisted observations are integrated into a unified observation framework to constrain the clock offset, frequency offset, and residual link term. In the state space, the residual bias and its rate of change are introduced to characterize the dominant time-varying behavior of the post-compensation residual asymmetric error within a short sampling interval. Furthermore, a link-state-information-assisted IMM-IEKF online estimation mechanism is adopted. Computable indicators such as maneuvering intensity and link-degradation degree are used to adjust mode priors and noise covariances, allowing the estimation process to adapt to changes in residual evolution and observation reliability under strong-dynamic and degraded-link conditions.

The main contributions of this paper are summarized as follows:

(1) Dynamic state representation is established for compensated residual asymmetric errors. This paper models the asymmetric link error that remains after geometric and motion compensation as a recursively estimable dynamic state, and it introduces its rate of change to describe the dominant time-varying features of the residual error within a short sampling interval. This provides a state-space basis for the explicit tracking of residual asymmetric errors under high-dynamic conditions.
(2) A four-timestamp–frequency-offset joint observation model is constructed to enhance state separability. This paper integrates compensated four-timestamp observations and frequency-offset-assisted observations into the same observation framework, extending frequency-offset information from state prediction or frequency-estimation assistance to an observation-level constraint. This alleviates the coupling between the clock-offset term and the residual link term, and it enables joint estimation of residual asymmetric errors and clock parameters.
(3) A link-state-information-assisted online estimation mechanism is implemented. This paper adopts IMM-IEKF as the online estimation framework and uses computable indicators such as maneuvering intensity and link-degradation degree to adjust the mode priors and noise covariance, allowing residual-state estimation to adapt to error evolution under strong-dynamic and degraded-link conditions.

The remainder of this paper is organized as follows. Section 2 introduces the joint observation and residual-state estimation method. Section 3 presents the simulation design. Section 4 provides the simulation results and analysis. Finally, this paper is summarized, and future research directions are discussed.

## 2. Joint Observation and Residual-State Estimation Method

### 2.1. High-Dynamic Bidirectional Microwave Link and Four-Timestamp Definition

A single-link microwave two-way time-synchronization system is considered, comprising a highly dynamic mobile node A and a ground reference node B. Node B acts as the reference clock node, and the clock parameters of node A are estimated through bidirectional message exchange [15,16]. In the k-th two-way exchange, node B first transmits an interrogation signal. Upon reception, node A sends a response signal after a local processing delay, which is then received by node B.

For simplicity, the four local timestamps are denoted as $T_{1,k}$, $T_{2,k}$, $T_{3,k}$, and $T_{4,k}$, which correspond to the transmission time at node B, the reception time at node A, the response transmission time at node A, and the response reception time at node B, respectively. Specifically, $T_{1,k}$ and $T_{4,k}$ are recorded by the clock of node B, and $T_{2,k}$ and $T_{3,k}$ are recorded by the clock of node A. Therefore, although these four timestamps describe the same two-way message exchange, they are not measured on the same local time scale.

Taking the time scale of node B as the reference, the clock offset of node A relative to node B is denoted by $\theta_{k}$, the relative frequency offset by $y_{k}$, and the frequency drift by ${\dot{y}}_{k}$. Within a short sampling interval, the clock of node A can be locally approximated as follows [6,17]:

(1) $$C_{A}(t)\approx C_{B}(t)+\theta_{k}+y_{k}(t-t_{k})+\frac{1}{2}{\dot{y}}_{k}{\left(t-t_{k}\right)}^{2}$$

Here, $C_{A}(t)$ and $C_{B}(t)$ denote the clock readings of nodes A and B at physical time t, respectively, and $t_{k}$ denotes the reference time of the k-th estimation epoch. In this paper, the clock offset is defined as follows:

(2) $$\theta_{k}=C_{A}(t_{k})-C_{B}(t_{k})$$

Let $\tau_{BA,k}$ and $\tau_{AB,k}$ denote the propagation delays in the B-to-A and A-to-B directions, respectively, and let $T_{turn,k}$ denote the local turnaround delay at node A [17,18]. Since the duration of a single two-way exchange is much shorter than the state update interval, the effects of the frequency offset and frequency drift on individual timestamps can be absorbed into equivalent errors and explicitly modeled later through frequency-offset observations and state propagation. The timestamp relationships can then be written as follows [6,18]:

(3) $$T_{2,k}=T_{1,k}+\tau_{BA,k}+\theta_{k}+\eta_{2,k}$$

(4) $$T_{3,k}=T_{2,k}+T_{turn,k}+\eta_{3,k}$$

(5) $$T_{4,k}=T_{3,k}+\tau_{AB,k}-\theta_{k}+\eta_{4,k}$$

Here, $\eta_{2,k}$ and $\eta_{4,k}$ denote the reception timestamp errors at nodes A and B, respectively, and $\eta_{3,k}$ denotes the uncertainty associated with the local turnaround delay at node A. Since $\theta_{k}=C_{A}-C_{B}$, converting the B-side time scale to the A-side time scale introduces $+\theta_{k}$, whereas converting the A-side time scale back to the B-side time scale introduces $-\theta_{k}$.

Under static or low-dynamic conditions, the bidirectional propagation paths typically satisfy $\tau_{BA,k}\approx \tau_{AB,k}$. However, under highly dynamic conditions, due to the relative motion of the platforms, continuous variations in propagation geometry, and the influence of the Doppler effect, this approximation no longer holds, and the two propagation delays cannot be effectively canceled [4].

Furthermore, factors such as link noise fluctuations, local tracking errors, and attitude disturbances further amplify this asymmetry. As a result, the asymmetry exhibits mode-switching behavior and local abrupt variations over time, thereby becoming one of the primary sources of errors in highly dynamic TWTT systems.

### 2.2. Four-Timestamp Observation and Post-Compensation Residual Bias

Based on the four timestamps, a conventional four-timestamp clock-offset observation can be constructed as follows [17,18]:

(6) $$z_{\theta ,k}^{\left(4T\right)}=\frac{\left(T_{2,k}-T_{1,k})-(T_{4,k}-T_{3,k}\right)}{2}$$

Substituting Equations (3) and (5) into Equation (6), the raw four-timestamp observation can be obtained as follows:

(7) $$z_{\theta ,k}^{\left(4T\right)}=\theta_{k}+\frac{\tau_{BA,k}-\tau_{AB,k}}{2}+\nu_{\theta ,k}$$

where $\nu_{\theta ,k}$ denotes the equivalent observation noise after the combination of the four timestamps.

From Equation (7), it can be seen that, in highly dynamic bidirectional microwave links, the conventional four-timestamp observation not only contains the clock offset $\theta_{k}$ to be estimated but also explicitly couples the propagation-delay asymmetry term [6,19]. This term is defined as follows:

(8) $$b_{k}^{asym}=\frac{1}{2}(\tau_{BA,k}-\tau_{AB,k})$$

This term represents the equivalent bias introduced by uplink–downlink propagation asymmetry in highly dynamic bidirectional microwave links. Accordingly, Equation (6) can be expressed as follows:

(9) $$z_{\theta ,k}^{\left(4T\right)}=\theta_{k}+b_{k}^{asym}+\nu_{\theta ,k}$$

In high-dynamic scenarios, a geometric and motion compensation term can be calculated using trajectory, velocity, and propagation models. Let this compensation term be denoted by ${\hat{b}}_{k}^{geom}$. The compensated four-timestamp observation is then given as follows:

(10) $$z_{\theta ,k}^{\left(gc\right)}=z_{\theta ,k}^{\left(4T\right)}-{\hat{b}}_{k}^{geom}=\theta_{k}+b_{k}+\nu_{\theta ,k}$$

where $b_{k}=b_{k}^{asym}-{\hat{b}}_{k}^{geom}$ denotes the residual asymmetric dynamic error that remains after geometric-motion compensation. This residual bias cannot be adequately represented as either white noise or a fixed bias, because it originates from highly dynamic propagation modeling errors, local compensation mismatch, small-scale propagation disturbances, and link degradation [13]. Moreover, it often exhibits pronounced time-varying and mode-switching behavior during periods of intense maneuvering and degradation. Therefore, the main challenge addressed in this paper is not merely the construction of geometric compensation terms but also the explicit modeling and online estimation of the residual bias $b_{k}$ after compensation.

Equation (10) shows that the clock offset $\theta_{k}$ and the residual link bias $b_{k}$ enter the compensated four-timestamp observation in an additive form. Under a single-link condition, these two terms are difficult to separate sufficiently. Additional observation constraints are therefore required to improve their separability.

### 2.3. Frequency-Offset-Aided Observation and Joint Observation Model

As can be seen from the observation relationship given by Equation (10), under single-link conditions, the clock offset $\theta_{k}$ and residual bias $b_{k}$ are coupled in an additive form in the observation equation, resulting in limited separability. To enhance the distinguishability between the clock term and the link residual term under highly dynamic conditions, uplink and downlink frequency-offset-aided observations are further introduced [6,12].

Let the nominal carrier frequency of the link be denoted by $f_{c}$. The local oscillator frequency of node A relative to node B can be expressed as follows:

(11) $$f_{A,k}=f_{c}(1+y_{k})$$

For a high-dynamic bidirectional link, the frequency-offset observation contains not only the oscillator frequency difference but also the frequency response induced by the time variation of the propagation delay. After direction unification and nominal Doppler compensation, the remaining frequency-offset component may still include residual information caused by compensation errors, non-reciprocal propagation variations, and the local evolution of the post-compensation asymmetric residual [4,20].

To avoid interpreting the frequency-offset auxiliary quantity as a direct measurement of the residual bias, it is treated here as a local linear auxiliary constraint. Let ${\tilde{y}}_{BA,k}$ and ${\tilde{y}}_{AB,k}$ denote the uplink and downlink frequency-offset observations after direction unification and nominal Doppler compensation. The differential combination is mainly used to constrain the relative frequency offset and is expressed as follows:

(12) $$z_{y,k}=\frac{1}{2}({\tilde{y}}_{BA,k}-{\tilde{y}}_{AB,k})y_{k}+\nu_{y,k}$$

where $\nu_{y,k}$ denotes the noise of the differential frequency-offset observation. This observation mainly provides a direct constraint on the relative frequency offset $y_{k}$.

In addition, the summation-type combination of the uplink and downlink frequency-offset observations contains information related to propagation-geometry variation, Doppler asymmetry, and residual evolution [21]. It should be emphasized that this summation-type frequency-offset quantity is not a direct measurement of the residual bias $b_{k}$. Instead, it provides an auxiliary constraint associated with the residual state. After local linearization around the current operating point, this auxiliary observation can be written as follows:

(13) $$z_{b,k}\approx \alpha_{k}b_{k}+\beta_{k}{\dot{b}}_{k}+\nu_{b,k}$$

Here, $\alpha_{k}$ and $\beta_{k}$ are local sensitivity coefficients determined by the current link geometry, the nominal Doppler compensation model, the frequency-offset construction, and the sampling interval. In the simulation, they are computed from the trajectory, velocity, and propagation-compensation model. In practical systems, they can be updated online using navigation-aided link-state information and local linearization. It should be emphasized that $z_{b,k}$ is not a direct measurement of $b_{k}$. Instead, it provides an auxiliary observation constraint related to the residual bias and its rate of change. Its role is to provide additional information for the combined term $\theta_{k}+b_{k}$ in the compensated four-timestamp observation, thereby improving the local separability between the clock state and the residual link state. $v_{b,k}$ denotes the noise of the summation-type frequency-offset auxiliary observation.

Combining Equations (10), (12), and (13), the joint observation vector is constructed as follows:

(14) $$z_{k}=\left[\begin{array}{c} z_{\theta ,k}^{\left(gc\right)}\\ z_{y,k}\\ z_{b,k} \end{array}\right]\approx \left[\begin{array}{c} \theta_{k}+b_{k}\\ y_{k}\\ \alpha_{k}b_{k}+\beta_{k}{\dot{b}}_{k} \end{array}\right]+v_{k}$$

where the observation noise vector is given as follows:

(15) $$v_{k}={\left[\begin{array}{ccc} v_{\theta ,k} & v_{y,k} & v_{b,k} \end{array}\right]}^{T}$$

Equation (14) indicates that the compensated four-timestamp observation provides a combined constraint on the clock offset and the residual link bias, the differential frequency-offset observation constrains the relative frequency offset, and the summation-type frequency-offset auxiliary observation provides an additional constraint on the residual bias and its rate of change. Together, these observations form the four-timestamp–frequency-offset joint observation model.

### 2.4. Residual-State Model and Local Observability Analysis

To recursively estimate the post-compensation residual asymmetric error, the clock offset, relative frequency offset, frequency drift, residual bias, and residual-bias rate are incorporated into a unified state vector as follows:

(16) $$x_{k}={\left[\begin{array}{ccccc} \theta_{k} & y_{k} & {\dot{y}}_{k} & b_{k} & {\dot{b}}_{k} \end{array}\right]}^{T}$$

Here, $b_{k}$ and ${\dot{b}}_{k}$ are used to characterize the low-dimensional time-varying behavior of the post-compensation residual asymmetric error. This first-order residual model is not intended to precisely describe all nonlinear or abrupt disturbances. Instead, it captures the dominant residual dynamics within a short sampling interval. The uncertainty caused by strong maneuvering, model mismatch, and link degradation is handled later through mode switching and adaptive noise adjustment.

Within the sampling interval Δt, the state transition model is given as follows:

(17) $$x_{k+1}=Fx_{k}+w_{k}$$

where **F** is the discrete-time state-transition matrix, and $w_{k}∼N(0,Q_{k})$ denotes the process noise. The corresponding state-transition matrix is expressed as follows:

(18) $$F=\left[\begin{array}{ccccc} 1 & \Delta t & \frac{1}{2}\Delta t^{2} & 0 & 0\\ 0 & 1 & \Delta t & 0 & 0\\ 0 & 0 & 1 & 0 & 0\\ 0 & 0 & 0 & 1 & \Delta t\\ 0 & 0 & 0 & 0 & 1 \end{array}\right]$$

Using the joint observation in Equation (14), the observation model can be written as follows:

(19) $$z_{k}=h(x_{k})+v_{k}$$

where the nonlinear observation function is given as follows:

(20) $$h(x_{k})=\left[\begin{array}{c} \theta_{k}+b_{k}\\ y_{k}\\ \alpha_{k}b_{k}+\beta_{k}{\dot{b}}_{k} \end{array}\right]$$

To illustrate the role of frequency-offset-aided observations in improving state separability, the local observability of two observation structures is compared [19,21]. When only the compensated four-timestamp observation is used, the local linear observation matrix is given as follows:

(21) $$H_{0}=\left[\begin{array}{ccccc} 1 & 0 & 0 & 1 & 0 \end{array}\right]$$

The corresponding local observability matrix is defined as follows:

(22) $$O_{0}=\left[\begin{array}{c} H_{0}\\ H_{0}F_{k}\\ H_{0}F_{k}^{2}\\ H_{0}F_{k}^{3}\\ H_{0}F_{k}^{4} \end{array}\right]$$

Under the condition Δt≠0, its rank satisfies the following relation:

(23) $$rank(O_{0})=3<5$$

This result indicates that, with only the compensated four-timestamp observation, the system mainly observes the combined quantity $\theta_{k}+b_{k}$. Although the state vector includes both the clock offset and the residual-bias state, the two terms enter the four-timestamp observation in an additive form. Therefore, the clock offset and the residual link bias cannot be sufficiently separated under a single-link four-timestamp-only observation structure.

After introducing the frequency-offset-aided observations, the local linearized observation matrix is given as follows:

(24) $$H=\left[\begin{array}{ccccc} 1 & 0 & 0 & 1 & 0\\ 0 & 1 & 0 & 0 & 0\\ 0 & 0 & 0 & \alpha_{k} & \beta_{k} \end{array}\right]$$

The corresponding local observability matrix is defined as follows:

(25) $$O_{J}=\left[\begin{array}{c} H_{J}\\ H_{J}F_{k}\\ H_{J}F_{k}^{2}\\ H_{J}F_{k}^{3}\\ H_{J}F_{k}^{4} \end{array}\right]$$

Under the local constant-coefficient approximation of $\alpha_{k}$ and $\beta_{k}$ within the observability window, a sufficient non-degenerate condition for full local observability is: Δt≠0 and $\alpha_{k}\neq 0$. This condition can be verified by selecting five independent rows from $O_{J}$. The determinant of the corresponding submatrix is proportional to $\alpha_{k}^{2}\Delta t^{2}$.

Therefore, when Δt≠0 and the auxiliary frequency-offset observation has non-zero local sensitivity to the residual-bias state $b_{k}$, the rank condition becomes the following:

(26) $$rank(O_{J})=5$$

Therefore, under the above non-degenerate condition, the joint observation structure provides additional locally independent constraints for the clock offset, relative frequency offset, and residual link states. However, the rank condition only reflects structural local observability and does not fully characterize numerical observability or noise sensitivity. To reduce the influence of different state units and numerical scales, the local observability matrix is first column-normalized, and its minimum singular value and condition number are then used as numerical indicators:

(27) $$\sigma_{\min}(O)$$

(28) $$\mathrm{cond}(O)=\frac{\sigma_{\max}(O)}{\sigma_{\min}(O)}$$

A larger minimum singular value and a smaller condition number indicate better numerical observability. In addition, the posterior correlation between $\theta_{k}$ and $b_{k}$, together with noise-sensitivity results, will be evaluated in Section 4 to further verify the separability improvement provided by the joint observation structure.

### 2.5. Link-State-Information-Assisted IMM-IEKF

The post-compensation residual asymmetric error varies with platform maneuvering, propagation-geometry change, and link quality. To adapt to such variations, this paper adopts a link-state-informed multi-model online estimation mechanism. The modes are not treated as strict physical models. Instead, they are defined as representative estimation states associated with computable link-state indicators [22,23,24,25]. Three representative modes are defined as follows:

Normal Mode M_1_: The normal mode corresponds to relatively smooth propagation-geometry variations and stable observation quality.

Strong-Dynamic Mode M_2_: The high-dynamic mode corresponds to enhanced platform maneuvering or rapid propagation-geometry variation.

Degradation Mode M_3_: The degraded mode corresponds to link-quality deterioration, increased observation noise, or reduced observation reliability.

To establish a quantitative connection between the modes and link states, the maneuvering-intensity score $s_{man,k}$ and the degradation-severity score $s_{\deg,k}$ are introduced. The maneuvering-intensity score is constructed as follows:

(29) $$s_{man,k}=sat_{\left[0,1\right]}(w_{a}\frac{\left|a_{r,k}\right|}{a_{\mathrm{ref}}}+w_{D}\frac{\left|{\dot{f}}_{D,k}\right|}{{\dot{f}}_{\mathrm{ref}}})$$

The degradation-severity score is constructed as follows:

(30) $$s_{\deg,k}=sat_{\left[0,1\right]}(w_{\varepsilon }\frac{{\bar{\varepsilon }}_{k}}{\chi_{m}^{2}(p)}+w_{m}r_{\mathrm{miss},k}+w_{q}q_{\mathrm{link},k})$$

Here, $a_{r,k}$ denotes the radial acceleration, ${\dot{f}}_{D,k}$ denotes the Doppler-rate variation, $a_{\mathrm{ref}}$ and ${\dot{f}}_{D,\mathrm{ref}}$ are normalization reference values, ${\bar{\varepsilon }}_{k}$ denotes the normalized innovation squared or its smoothed value, $r_{\mathrm{miss},k}$ denotes the missing-observation ratio, $q_{\mathrm{link},k}$ denotes the link-quality degradation indicator, and ${\mathrm{sat}}_{\left[0,1\right]}(\cdot )$ restricts the result to the interval [0, 1].

Let the IMM transition probability matrix be defined as follows:

(31) $$\Pi =\left[\pi_{ij}\right]\;i,j=1,2,3$$

Given the posterior mode probability $\mu_{k-1}^{\left(i\right)}$ at the previous epoch, the conventional transition-predicted mode probability is computed as follows:

(32) $${\bar{\mu }}_{k|k-1}^{\left(j\right)}=\sum_{i=1}^{3}\pi_{ij}\mu_{k-1}^{\left(i\right)}$$

The mode prior is then modified using the link-state scores. The link-state mode weights are defined as follows:

(33) $$g_{1,k}=(1-s_{\mathrm{man},k})(1-s_{\deg,k})$$

(34) $$g_{2,k}=s_{\mathrm{man},k}(1-s_{\deg,k})$$

(35) $$g_{3,k}=s_{\deg,k}$$

The modified mode prior is obtained as follows:

(36) $$\mu_{k|k-1}^{\left(j\right)}=\frac{{\bar{\mu }}_{k|k-1}^{\left(j\right)}(g_{j,k}+g_{\min})}{\sum_{l=1}^{3}{\bar{\mu }}_{k|k-1}^{\left(l\right)}(g_{l,k}+g_{\min})}$$

Here, $g_{\min}$ is a small positive constant used to prevent mode-probability degeneration.

In each mode, an IEKF is used for state updating, while the process noise and observation noise are adjusted according to the link state [19,26]. The observation-noise inflation factor is defined as follows:

(37) $$\kappa_{R,k}^{\left(j\right)}={\mathrm{clip}}_{\left[\kappa_{R}^{\min},\kappa_{R}^{\max}\right]}\left[1+\lambda_{\varepsilon }\max(0,\frac{\varepsilon_{k}^{\left(j\right)}}{\chi_{m}^{2}(p)}-1)+\lambda_{d}s_{\deg,k}+\lambda_{m}s_{\mathrm{miss},k}\right]$$

The process-noise inflation factor is defined as follows:

(38) $$\kappa_{Q,k}^{\left(j\right)}={\mathrm{clip}}_{\left[\kappa_{Q}^{\min},\kappa_{Q}^{\max}\right]}\left[1+\rho_{m}s_{man,k}+\rho_{d}s_{\deg,k}\right]$$

Thus, the mode-dependent noise covariance matrices are updated as follows:

(39) $$R_{k}^{\left(j\right)}=\kappa_{R,k}^{\left(j\right)}R_{0}^{\left(j\right)},\;Q_{k}^{\left(j\right)}=\kappa_{Q,k}^{\left(j\right)}Q_{0}^{\left(j\right)}$$

Equation (37) improves the tolerance to innovation anomalies, missing observations, and link degradation. Equation (38) increases the state-evolution uncertainty under strong dynamics or link degradation.

The mode-conditioned IEKF follows the standard prediction, linearization, gain calculation, and state-correction steps. To control the computational cost for online implementation, the iteration is terminated according to the following criterion:

(40) $$\frac{\left\|{\hat{x}}_{k}^{\left(j,l+1\right)}-{\hat{x}}_{k}^{\left(j,l\right)}\right\|}{\left\|{\hat{x}}_{k}^{\left(j,l\right)}\right\|+\varepsilon_{0}}<\varepsilon_{\mathrm{IEKF}}\;or\;l=I_{\max}$$

Here, the convergence threshold is set to $\varepsilon_{\mathrm{IEKF}}=10^{-3}$, and the maximum number of iterations is set to $I_{\max}=3$. This setting limits the computational burden of each mode-conditioned update while allowing local refinement when the residual-domain linearization is insufficient.

After the mode-conditioned updates are completed, the posterior mode probability is updated according to the mode likelihood $\Lambda_{k}^{\left(j\right)}$ as follows:

(41) $$\mu_{k}^{\left(j\right)}=\frac{\Lambda_{k}^{\left(j\right)}\mu_{k|k-1}^{\left(j\right)}}{\sum_{l=1}^{3}\Lambda_{k}^{\left(l\right)}\mu_{k|k-1}^{\left(l\right)}}$$

The final fused state estimate is obtained as follows:

(42) $${\hat{x}}_{k|k}=\sum_{j=1}^{3}\mu_{k}^{\left(j\right)}{\hat{x}}_{k|k}^{\left(j\right)}$$

The final estimate is therefore not the output of a single mode but a probability-weighted fusion of estimates under different representative link states. The mode probabilities reflect both statistical model matching and the current link condition, indicating whether the link is closer to a normal, strong-dynamic, or degraded state.

## 3. Simulation Design and Evaluation Metrics

Because publicly available field-test data for high-dynamic two-way microwave time synchronization are limited, complex operating conditions such as strong maneuvers, link degradation, and enhanced observation noise are also difficult to reproduce systematically in field experiments. Therefore, this study validates the proposed method using a unified simulation platform. Under the same trajectory inputs, true clock parameters, propagation model, observation-noise injection strategy, and link-degradation settings, different methods are compared fairly. The compared methods differ only in observation modeling, residual-state modeling, and online estimation mechanisms. Thus, the simulation results can more directly reflect the effects of frequency-offset-assisted observation, residual-bias state augmentation, and the link-state-information-assisted IMM-IEKF on synchronization performance.

### 3.1. Simulation Scenario Setup

In the simulation, node A is a high-dynamic mobile node, while node B serves as the reference node and provides a stable time reference. Four-timestamp exchange and frequency-offset observation are performed through the two-way microwave link between nodes A and B. The total simulation duration is 2000 s, with a sampling interval of 0.02 s. To cover different dynamic intensities and link states, this study designs a layered set of scenarios, including nominal high-dynamic, strong-dynamic, noise-enhanced, degraded-link, composite-disturbance, and extreme high-dynamic scenarios, as shown in Table 1.

It should be noted that the strong-dynamic intervals and degraded-link intervals in this study are predefined according to the scenario configuration, rather than selected retrospectively based on the estimation results. The strong-dynamic intervals correspond to phases with rapid variations in velocity, acceleration, and Doppler-related terms. The degraded-link intervals correspond to phases with reduced observation quality, increased measurement noise, and a lower proportion of valid observations.

### 3.2. Compared Methods and Residual Model Settings

To validate the effectiveness of the proposed method and analyze the role of each component, this study establishes a comparison framework ranging from baseline methods to the complete proposed method. All methods use the same trajectory inputs, true clock parameters, link propagation model, noise injection strategy, and evaluation metrics. They differ only in observation modeling, state modeling, and online estimation mechanisms. The specific algorithms are listed in Table 2.

Among them, GC4TS denotes the four-timestamp baseline method after geometric propagation compensation. FA-noBias is used to examine the performance obtained by introducing frequency-offset-assisted observations without explicitly estimating the residual bias. Robust-IEKF-RB and VB-IEKF-RB serve as robust filtering and adaptive Bayesian filtering baselines, respectively, and are used to compare single-model residual-bias recursive estimation methods with the proposed multi-model method [27,28]. IMM-RB denotes the proposed link-state-information-assisted residual-bias estimation method.

### 3.3. Experimental Organization and Evaluation Metrics

The experimental validation is organized from global performance to local diagnostics and from performance evaluation to mechanism analysis. The overall clock-offset estimation accuracy of different methods is first compared across multiple scenarios. The local error behavior is then examined within the strong-dynamic and degraded-link windows. Subsequently, Monte Carlo experiments, NIS indicators, component ablation, residual-bias tracking, and local separability analysis are used to verify the underlying mechanisms of the proposed method. Finally, mode response, parameter sensitivity, and per-epoch runtime are analyzed to evaluate the online adaptability and engineering feasibility of the proposed method.

To improve the reproducibility of the simulation experiments, the key parameters of the equivalent stochastic-error model and the estimator are summarized in Table 3. It should be noted that this study adopts an equivalent observation-noise and link-degradation model for algorithm validation rather than a complete physical-layer channel model. The parameters listed in the table are used to define the standard deviations of random noises, noise amplification factors, missing-observation probabilities, the residual-bias generation process, and the main adjustment parameters of the estimator.

This study evaluates different methods in terms of synchronization accuracy, robustness in key intervals, residual-bias estimation, local numerical separability, random-seed stability, and real-time computational feasibility. The main metrics include the overall clock-offset RMSE, RMSE in the strong-dynamic and degraded-link windows, peak moving RMSE, maximum absolute error, valid-output ratio, residual-bias RMSE, local observability indicators, NIS consistency, and real-time factor. Their definitions are given in Table 4, and the corresponding results and analyses are presented in Section 4.

In Table 4, W denotes a pre-defined critical interval, including the strong-dynamic interval or the degraded-link interval. $N_{W}$ is the number of epochs in W, and $N_{\mathrm{valid}}$ is the number of valid estimates. O denotes the local observability matrix. $\nu_{k}$ and $S_{k}$ denote the innovation vector and innovation covariance, respectively. ${\bar{t}}_{ep}$ is the average runtime per epoch, and Δt=20 ms.

## 4. Simulation Results and Validation Analysis

### 4.1. Synchronization Performance Under High-Dynamic and Degraded-Link Conditions

**Figure 1:**
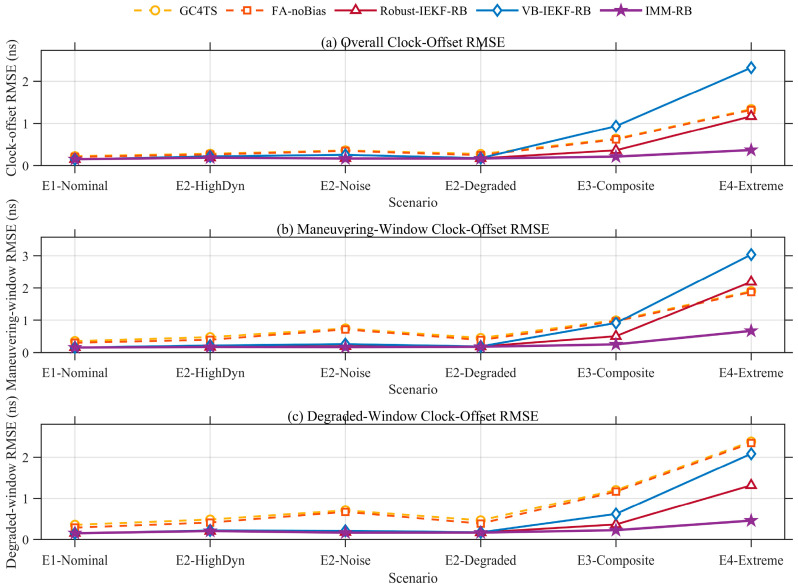
Multi-scenario comparison of clock-offset estimation performance. (**a**) Overall clock-offset RMSE over the full simulation interval; (**b**) clock-offset RMSE in the pre-defined maneuvering interval; (**c**) clock-offset RMSE in the pre-defined degraded-link interval.

To verify the synchronization performance of the proposed method under different high-dynamic link conditions, this study compares the clock-offset estimation errors of GC4TS, FA-noBias, Robust-IEKF-RB, VB-IEKF-RB, and IMM-RB across multiple scenarios. Figure 1 presents the comparison results of the overall clock-offset RMSE, the clock-offset RMSE in the strong-dynamic window, and the clock-offset RMSE in the degraded-link window. In relatively mild or single-disturbance scenarios, including E1-Nominal, E2-HighDynamic, E2-NoiseEnhanced, and E2-DegradedLink, the differences among the methods are relatively limited. This indicates that geometric compensation and frequency-offset assistance can still maintain a certain level of synchronization accuracy when the dynamic intensity is low or the compensated residual is weak. As the scenario’s complexity increases, especially in the E3-Composite and E4-Extreme scenarios, the errors of GC4TS and FA-noBias increase significantly. This suggests that relying only on geometric compensation or introducing frequency-offset assistance without explicitly estimating the residual bias is insufficient to suppress the residual asymmetric error caused by the combined effects of strong dynamic propagation variations and link degradation. In contrast, IMM-RB maintains relatively low errors across the three evaluation metrics, showing better adaptability under complex operating conditions.

**Figure 2:**
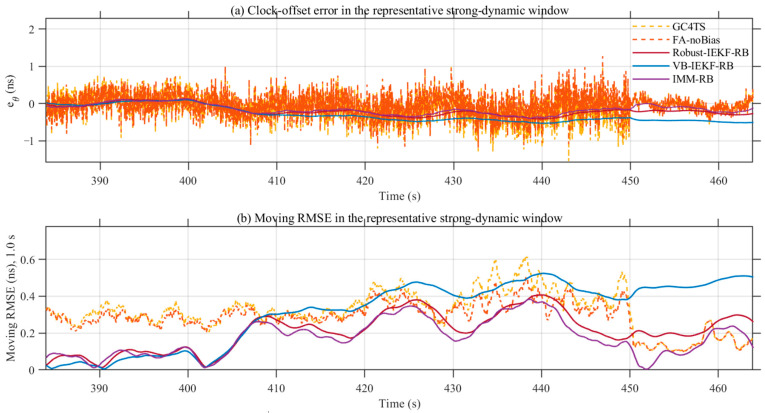
Clock-offset estimation performance in the strong-dynamic window. (**a**) Clock-offset estimation error $e_{\theta }$ of different methods; (**b**) the corresponding 1.0 s moving RMSE of $e_{\theta }$.

The representative strong-dynamic and degraded-link windows are further selected to analyze the local error behavior observed in the multi-scenario results. Figure 2 presents the clock-offset error and the 1.0 s moving RMSE within a representative strong-dynamic window. This window mainly reflects the influence of rapid propagation-geometry variation and fast residual-bias evolution on clock-offset estimation. GC4TS and FA-noBias exhibit more pronounced instantaneous error fluctuations, indicating that the post-compensation residual term can still directly affect clock-offset estimation. In contrast, the error curves of Robust-IEKF-RB, VB-IEKF-RB, and IMM-RB are generally smoother, suggesting that residual-bias-assisted filtering can suppress local error fluctuations under strong-dynamic conditions.

The quantitative results in Table 5 further show that IMM-RB achieves a window RMSE of 0.2175 ns, which is lower than those of GC4TS, FA-noBias, Robust-IEKF-RB, and VB-IEKF-RB. Its maximum moving RMSE and maximum absolute clock-offset error are 0.3819 ns and 0.3936 ns, respectively, both of which are the lowest among all methods. Compared with GC4TS, IMM-RB reduces the window RMSE by approximately 37.8% and the maximum absolute clock-offset error by approximately 68.4%. This indicates that IMM-RB has stronger adaptability to rapidly varying residual errors and better capability for suppressing transient peak errors in the strong-dynamic window.

**Figure 3:**
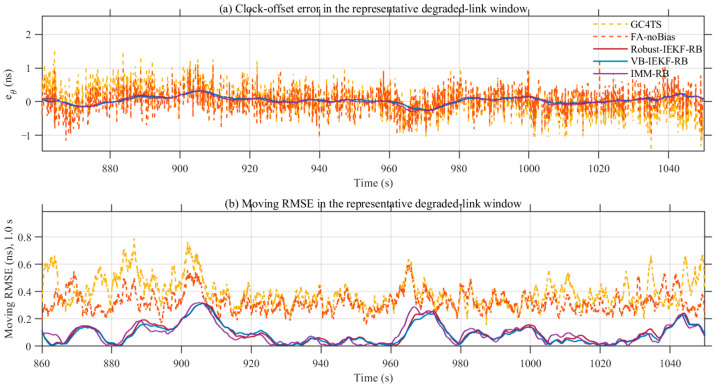
Clock-offset estimation performance in the degraded-link window. (**a**) Clock-offset estimation error $e_{\theta }$ of different methods; (**b**) the corresponding 1.0 s moving RMSE of $e_{\theta }$.

The error mechanism in the degraded-link window differs from that in the strong-dynamic window. It mainly reflects the influence of reduced observation quality, increased observation noise, and locally invalid observations on the estimation results. Figure 3 presents the clock-offset error and moving RMSE in the degraded-link window. GC4TS and FA-noBias show obvious local error peaks in this window, and their moving RMSE remains at a relatively high level for a long period. In comparison, all three residual-bias-assisted filtering methods significantly reduce error fluctuations, among which IMM-RB achieves the lowest overall moving RMSE curve and smaller local peaks.

Table 6 shows that the valid-output ratios of GC4TS and FA-noBias are both 0.7794, indicating that local missing observations and unavailable measurements directly affect non-recursive methods that rely on complete observation construction. Robust-IEKF-RB, VB-IEKF-RB, and IMM-RB all maintain valid-output ratios of 1.0000, showing that recursive filtering structures can preserve continuous valid estimation under degraded-link conditions. In terms of error metrics, IMM-RB achieves a window RMSE of 0.1421 ns, and its maximum moving RMSE and maximum absolute clock-offset error are 0.3166 ns and 0.3209 ns, respectively, all of which are the lowest in this window. Compared with GC4TS, IMM-RB reduces the window RMSE by approximately 64.1% and the maximum absolute clock-offset error by approximately 81.7%. This demonstrates that the link-state-information-assisted mode adjustment and observation-noise adaptation mechanism can effectively reduce the influence of degraded observations on clock-offset estimation.

**Figure 4:**
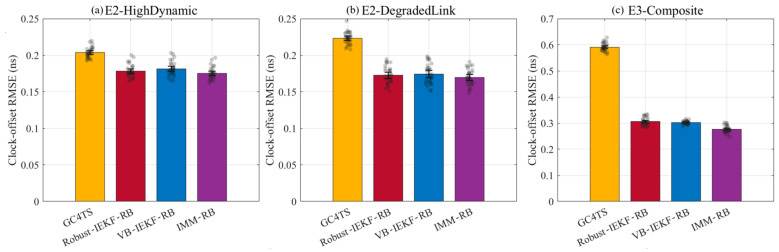
Monte Carlo robustness of clock-offset estimation under different random seeds. (**a**) E2-HighDynamic scenario; (**b**) E2-DegradedLink scenario; (**c**) E3-Composite scenario. The scattered points denote individual simulation results, and the black error bars indicate the variability of the repeated simulations.

To examine whether the above results depend on a single random-noise realization, 30 Monte Carlo experiments with different random seeds are conducted under three representative scenarios: E2-HighDynamic, E2-DegradedLink, and E3-Composite. In each experiment, the trajectory input, true clock parameters, propagation model, link-degradation window, and algorithm parameters are kept unchanged, while only the random seeds of the observation noise and process noise are varied. Figure 4 shows the clock-offset RMSE distributions under different random seeds. The results indicate that IMM-RB maintains a low average RMSE in all three scenarios, and its results are relatively concentrated across random seeds. In the E3-Composite scenario, the error of GC4TS increases significantly. Although Robust-IEKF-RB and VB-IEKF-RB improve the performance to some extent, IMM-RB still achieves the lowest average RMSE. This result shows that the performance advantage of IMM-RB is not caused by a specific noise realization but remains stable across multiple random disturbances.

To evaluate the statistical consistency of the filtering update process, this study further calculates the NIS metric of IMM-RB in the Monte Carlo experiments. NIS measures the consistency between the innovation sequence and the innovation covariance. Table 7 reports the Mean NIS and NIS 95% coverage under the three representative scenarios. The results show that IMM-RB does not exhibit obvious innovation divergence in these scenarios. In the E2-HighDynamic scenario, the NIS 95% coverage is 0.9042, which is closer to the theoretical coverage level, indicating that the innovation covariance is generally consistent with the actual innovation statistics under strong-dynamic conditions with relatively controllable observation quality. The coverage values in E2-DegradedLink and E3-Composite are 0.8832 and 0.8829, respectively, which are lower than the theoretical reference. This indicates that the innovation covariance is not fully calibrated under degraded-link and composite-disturbance conditions. Nevertheless, the Mean NIS values remain finite, and no innovation divergence is observed, suggesting that IMM-RB maintains stable recursive estimation, while further covariance calibration is still needed under degraded-link and composite-disturbance conditions.

Overall, these results show that IMM-RB improves both accuracy and output continuity under strong-dynamic and degraded-link conditions. The Monte Carlo and NIS analyses further support its stability under random disturbances.

### 4.2. Verification of the Role of Residual Asymmetric Error Modeling

The results in Section 4.1 have shown that, under strong-dynamic and degraded-link conditions, the method without explicit estimation of residual asymmetric errors, namely FA-noBias, is prone to increased clock-offset errors. To further explain the source of this performance difference, this section verifies the explicit modeling of compensated residual asymmetric errors from four aspects, including component ablation, residual-bias tracking in key windows, residual-bias error statistics, and comparison among different residual models. The aim is to analyze the role of residual-bias state augmentation in high-dynamic two-way time synchronization.

**Figure 5:**
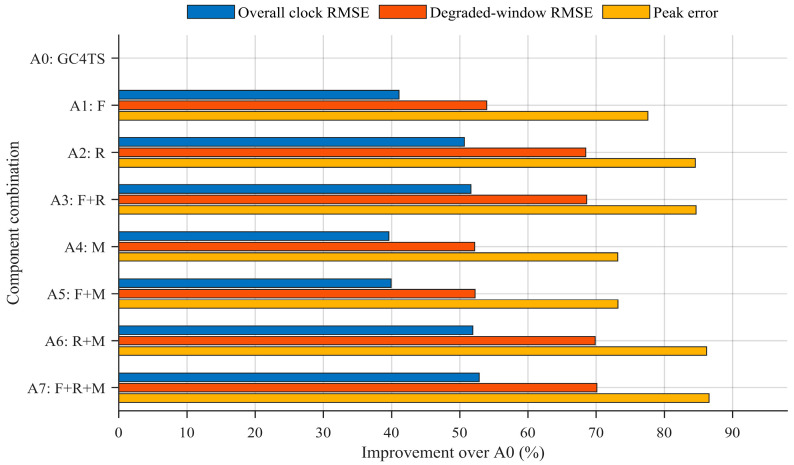
Component ablation results for frequency-offset assistance, residual-bias state augmentation, and IMM mode switching.

To distinguish the contributions of different modules to synchronization performance, this paper takes GC4TS as the baseline method A0 and constructs component ablation experiments based on three components: frequency-offset assistance, residual-bias state augmentation, and IMM mode switching. The ablation results are reported in terms of the improvement rate relative to A0. The evaluation metrics include the overall clock RMSE, degraded-window RMSE, and peak error. Figure 5 presents the ablation results of the three components.

The results in Figure 5 show that, after introducing residual-bias state augmentation alone, all three error metrics are significantly improved. This indicates that the compensated residual asymmetric error cannot be simply regarded as random noise or a fixed bias, but it has dynamic characteristics that need to be recursively estimated. Frequency-offset-assisted observation also brings certain improvements, but its main role is to provide additional observation constraints for separating the clock offset from the residual bias. When IMM mode switching is introduced alone, the degraded-window error and peak error are suppressed to some extent, indicating that the mode-switching mechanism can improve the estimator’s adaptability to changes in link state. The full combination of F + R + M achieves relatively balanced improvements in the overall clock RMSE, degraded-window RMSE, and peak error, suggesting that the performance improvement of the proposed method does not result from simple filter-parameter tuning but from the combined effects of joint observation, residual-state modeling, and link-state-information-assisted estimation.

**Figure 6:**
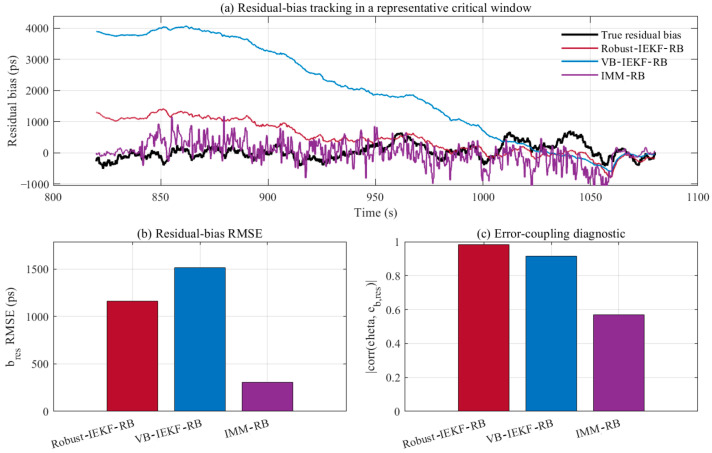
Residual-bias tracking and error-coupling diagnosis in a pre-defined critical interval. (**a**) Residual-bias trajectory and its estimates. The vertical axis denotes the residual bias bres itself in ps, rather than the residual-bias estimation error; (**b**) residual-bias estimation RMSE in the same interval; (**c**) error-coupling correlation between clock-offset error $e_{\theta }$ and residual-bias estimation error $e_{b,res}$.

To further examine whether the reduction in estimation error indeed corresponds to an improvement in residual-bias estimation capability, this paper selects a representative key window for residual-bias tracking and error-coupling diagnosis. The results are shown in Figure 6. Compared with the two single-model filtering baselines, Robust-IEKF-RB and VB-IEKF-RB, IMM-RB tracks the true residual bias more closely. In particular, in the first half of the window, VB-IEKF-RB clearly overestimates the residual bias, while Robust-IEKF-RB also shows a certain trend bias. Although IMM-RB still exhibits local fluctuations, its overall bias is smaller.

The residual-bias RMSE in Figure 6b further shows that IMM-RB achieves a significantly lower residual-bias estimation error than the two advanced single-model filtering baselines. Figure 6c presents the absolute correlation coefficient between the clock-offset error $e_{\theta }$ and the residual-bias estimation error $e_{b,res}$. The correlation coefficients of Robust-IEKF-RB and VB-IEKF-RB are close to 1, indicating that the clock-offset estimation error and the residual-bias estimation error remain highly coupled under the single-model filtering structure. In contrast, IMM-RB reduces this correlation to approximately 0.57, suggesting that the link-state-information-assisted multi-model structure can weaken the coupling between the clock-offset error and the residual-bias estimation error.

Table 8 further reports the residual-bias estimation errors of IMM-RB under different scenarios. RMSE(b_res_), MAE(b_res_), and P_95_(|e_b,res_|) denote the root mean square error, mean absolute error, and 95th percentile of the absolute residual-bias estimation error, respectively. RMSE_deg_(b_res_) denotes the residual-bias estimation error within the degraded-link intervals. It should be noted that these metrics are computed for $e_{b,res}={\hat{b}}_{res}-b_{res}$ rather than for the magnitude of b_res_ itself. The results indicate that strong dynamics and link degradation increase the difficulty of residual-bias estimation, but IMM-RB can still maintain bounded and quantifiable residual-bias estimation errors across different scenarios.

To verify the rationality of the residual model selected in this paper, different residual models are compared and analyzed. We compared a random-walk residual-bias model (RW-RB), a constant-rate residual-bias model (CR-RB), a higher-order residual-bias model (CA-RB), and an IMM-based mode-dependent constant-rate residual-bias model (IMM-CR-RB). The results show that simply increasing the model order does not necessarily improve clock-offset estimation performance. Compared with CR-RB, CA-RB reduces the residual-bias RMSE by approximately 2.5%, but it provides only limited improvement in the overall clock-offset RMSE and the degraded-window clock-offset RMSE. This indicates that the additional introduction of higher-order residual states does not introduce synchronization accuracy gains that are commensurate with the increased model complexity. In contrast, compared with CR-RB, IMM-CR-RB reduces the overall clock-offset RMSE, degraded-window clock-offset RMSE, and residual-bias RMSE by approximately 3.6%, 8.0%, and 6.4%, respectively. These results indicate that, under high-dynamic and degraded-link conditions, the link-state-information-assisted multi-model adaptation mechanism is more effective than simply increasing the residual-model order in improving residual-bias tracking and clock-offset estimation performance.

### 4.3. Separability and Robustness of the Joint Observation Structure

After the residual bias is introduced into the state vector, the key issue of the estimation problem is not only whether $b_{res}$ can be recursively estimated but also whether the clock-offset state θ and the residual-bias state $b_{res}$ can be stably distinguished at the observation level. For the observation structure using only compensated four timestamps, the observation mainly reflects the combined term $\theta_{k}+b_{res,k}$. Therefore, even if the residual-bias variable is introduced into the state equation, the clock-offset error and the residual-bias error may still be mixed in the local estimation process, leading to weak observability or numerical ill-conditioning in extended-state estimation.

**Figure 7:**
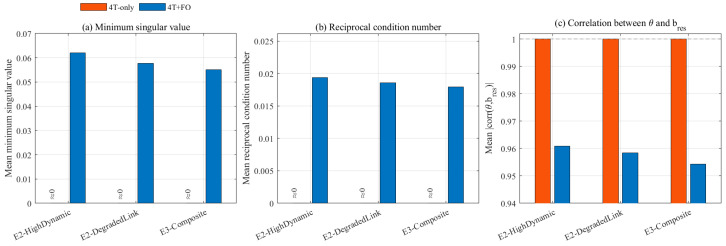
Local numerical observability comparison between the 4T-only and 4T + FO observation structures. (**a**) Mean minimum singular value of the local observability matrix; (**b**) mean reciprocal condition number of the local observability matrix; (**c**) mean correlation between the clock-offset state θ and the residual-bias state bres.

To verify the role of frequency-offset-assisted observations in extended-state estimation, this study compares two observation structures, namely the 4T-only structure and the 4T + FO structure. The former uses only compensated four-timestamp observations, whereas the latter further introduces frequency-offset observations. A sliding-window local observability matrix is constructed, and the minimum singular value, reciprocal condition number, and correlation between θ and $b_{res}$ are calculated to evaluate the local numerical separability of the two observation structures. The results are shown in Figure 7.

Figure 7 shows that, in the three representative scenarios, E2-HighDynamic, E2-DegradedLink, and E3-Composite, the minimum singular values and reciprocal condition numbers of the 4T-only structure are close to zero. This indicates that, when only compensated four-timestamp observations are used, the extended-state system has obviously weakly observable directions, and the local observation matrix is strongly ill-conditioned. In contrast, the minimum singular values of the 4T + FO structure are clearly greater than zero, and the reciprocal condition numbers remain within a finite range. This suggests that frequency-offset-assisted observations provide additional independent constraints for extended-state estimation and improve local numerical observability. The state-correlation results further support this conclusion. Under the 4T-only structure, the correlation between θ and $b_{res}$ is close to one, indicating that this structure mainly identifies their combined quantity and cannot sufficiently distinguish clock-offset variation from residual link-bias variation. After frequency-offset-assisted observations are introduced, the correlation decreases to approximately 0.95. Although the clock-offset state and residual-bias state are not completely decoupled, the 4T + FO structure reduces their high correlation in local estimation and improves the local separability of the extended state.

**Figure 8:**
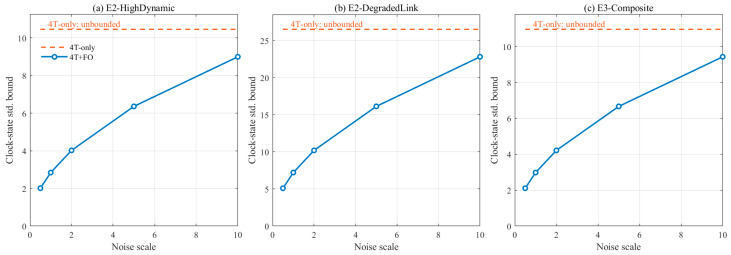
Comparison of the sensitivity of clock state uncertainty to noise under different observation structures. (**a**) E2-HighDynamic scenario; (**b**) E2-DegradedLink scenario; (**c**) E3-Composite scenario.

Extended-state estimation also needs to remain stable under enhanced-noise conditions. For this purpose, the clock-state standard deviation bound under different observation-noise scales is analyzed to characterize how the uncertainty of the clock-offset state changes as the noise level increases. The results are shown in Figure 8. After the residual-bias state is introduced, the 4T-only structure cannot provide a stable and finite uncertainty bound for the clock-offset state, and it is characterized as unbounded or rank-deficient. This indicates that, when only four-timestamp observations are used, introducing the residual-bias state aggravates the mixing between the clock-offset state and the residual link term, making the estimation problem more unstable under noisy conditions. In contrast, the 4T + FO structure maintains finite uncertainty bounds for the clock-offset state in the E2-HighDynamic, E2-DegradedLink, and E3-Composite scenarios, and these bounds increase smoothly with the noise scale. This indicates that frequency-offset-assisted observations not only improve local numerical observability but also reduce the sensitivity of extended-state estimation to observation noise.

Overall, the 4T-only structure tends to mix the clock offset and residual bias into a single combined quantity, leading to local ill-conditioning and increased noise sensitivity. By introducing frequency-offset-related constraints, the 4T + FO structure improves local separability and maintains a more stable state-uncertainty bound under enhanced-noise conditions. This provides an observation-structure-level explanation for the synchronization performance improvement in Section 4.1 and the residual-bias tracking improvement in Section 4.2.

### 4.4. Adaptability and Feasibility of Link-State-Information-Assisted Estimation

The preceding results show that residual-bias state modeling and the four-timestamp–frequency-offset joint observation can improve the estimation accuracy of high-dynamic two-way microwave time synchronization. This section further verifies the online adaptability and real-time feasibility of the link-state-information-assisted IMM-RB from three aspects: mode response, parameter sensitivity, and computational cost.

**Figure 9:**
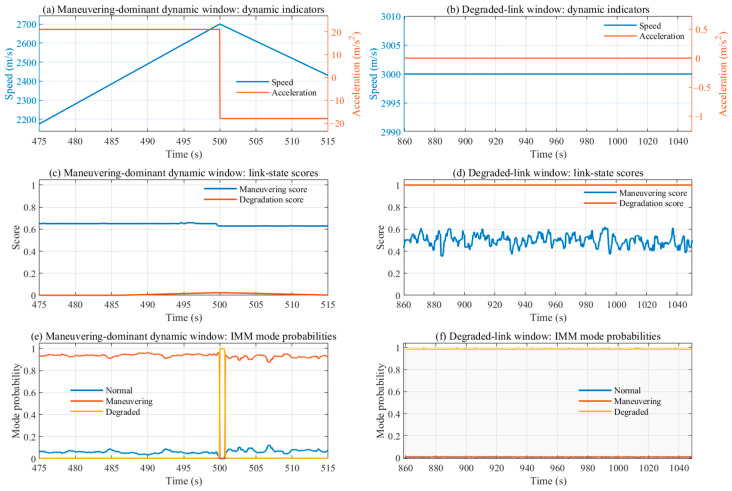
Link-state score response and IMM mode-probability adaptation in pre-defined critical intervals. (**a**) Dynamic indicators in the pre-defined strong-dynamic interval; (**b**) dynamic indicators in the pre-defined degraded-link interval; (**c**) link-state scores in the pre-defined strong-dynamic interval; (**d**) link-state scores in the pre-defined degraded-link interval; (**e**) IMM mode probabilities in the pre-defined strong-dynamic interval; (**f**) IMM mode probabilities in the pre-defined degraded-link interval.

A maneuvering-dominant dynamic window and a degraded-link window are selected for local analysis. The former mainly corresponds to enhanced relative motion, radial acceleration variation, and rapid changes in Doppler-related terms, whereas the latter mainly corresponds to reduced observation quality, increased degradation score, and enhanced measurement uncertainty. Figure 9 presents the dynamic indicators, link-state scores, and IMM mode probabilities. In the maneuvering-dominant dynamic window, the velocity and acceleration vary noticeably, the maneuvering score remains high, and the degradation score is close to zero. Correspondingly, the IMM mode probability is mainly concentrated in the maneuvering mode. In the degraded-link window, the velocity and acceleration remain generally stable, but the degradation score is close to one, and the IMM mode probability is almost entirely concentrated in the degraded mode. This result indicates that IMM-RB can distinguish the two main uncertainty sources, namely strong dynamics and observation degradation, according to link-state variations and adjust the mode probabilities accordingly.

To examine the influence of parameter selection on the results, a sensitivity analysis is conducted for the number of modes, the self-transition probability $p_{stay}$, the degraded-mode noise inflation factor $\lambda_{\deg}$, and the degradation-score threshold $\tau_{\deg}$. Since these parameters mainly affect link-degradation identification, mode-probability updating, and degraded-observation noise adjustment, the overall clock-offset RMSE and degraded-window RMSE are compared under the E2-DegradedLink and E3-Composite scenarios. The parameter ranges are set within engineering-reasonable intervals around the baseline values rather than extremely unstable ranges. Specifically, the number of modes ranges from 2 to 4, the self-transition probability ranges from 0.90 to 0.995, the degradation noise inflation factor ranges from 2 to 20, and the degradation-score threshold ranges from 0.3 to 0.7. These settings correspond to different mode complexities, mode persistence strengths, degradation-noise adjustment levels, and degradation-trigger sensitivities.

**Figure 10:**
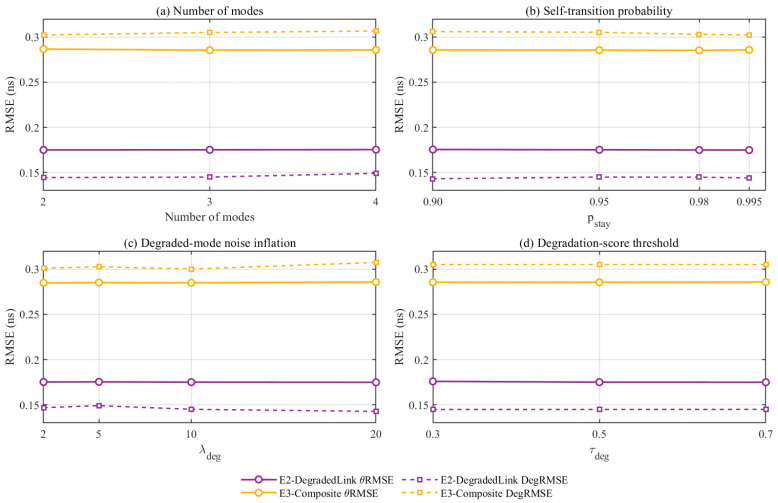
Sensitivity analysis of IMM mode-design parameters. (**a**) Number of modes N_m_; (**b**) self-transition probability p_stay_; (**c**) degraded-mode noise-inflation factor $\lambda_{\deg}$; (**d**) degradation-score threshold $\tau_{\deg}$.

The results in Figure 10 show that, within the tested ranges, the RMSE curves vary smoothly overall, without obvious instability or abrupt performance degradation. This indicates that the main performance conclusions of IMM-RB do not rely excessively on a single empirical parameter setting. This is because IMM-RB is not a hard-threshold switching mechanism. Its final estimate is jointly determined by the mode-transition prior, link-state score, innovation likelihood, and mode-probability fusion.

For an online time synchronization system, parameter stability alone is not sufficient; the single-epoch computational cost should also be evaluated. Table 9 reports the state dimension, number of modes, average single-epoch running time, real-time factor, and recovery time after link degradation for different methods. Here, ${\bar{t}}_{ep}$ denotes the average single-epoch running time of the online estimation loop, $\rho_{RT}$ denotes the real-time factor, and $T_{rec}$ denotes the estimation recovery time after the degraded link ends. GC4TS and FA-noBias are direct compensation-based methods and have the lowest computational cost, but they require longer recovery time after link degradation. Robust-IEKF-RB and VB-IEKF-RB are single-model recursive filtering methods, with average single-epoch running times of 0.1590 ms and 0.1830 ms, respectively. IMM-RB introduces interactions among three modes, probability updating, and mode fusion, increasing the average single-epoch running time to 0.6420 ms. Nevertheless, this value is still much lower than the 20 ms sampling interval. The corresponding real-time factor is $3.21\cdot 10^{-2}$, far below one, indicating that multi-model interaction and IEKF iteration do not introduce an unacceptable real-time computational burden.

Overall, IMM-RB not only shows good synchronization performance but also satisfies the basic requirements for online implementation. The link-state scores provide interpretable inputs for mode-probability adjustment, the parameter sensitivity results show that the main conclusions remain stable within reasonable parameter ranges, and the computational cost results further indicate that the proposed method can meet the real-time estimation requirement under the 20 ms sampling interval used in this study.

## 5. Discussion

The simulation results show that, in high-dynamic two-way microwave links, synchronization performance depends not only on whether the dominant propagation-asymmetry term can be compensated but also on whether the post-compensation residual asymmetric error can be effectively separated, characterized, and suppressed. Conventional four-timestamp methods and geometric compensation methods mainly address the dominant propagation term, but they lack explicit modeling of the post-compensation residual error. Therefore, error accumulation may occur under strong-dynamic and degraded-link conditions. FA-noBias introduces frequency-offset-assisted observations and can improve the observation constraints. However, since the residual bias is not included in the state space, its ability to continuously track the dynamic evolution of residual errors remains limited. Robust-IEKF-RB and VB-IEKF-RB further introduce the residual-bias state and improve adaptability to abnormal observations and noise variations through robust filtering or adaptive noise adjustment. Nevertheless, both methods still belong to single-model recursive estimation frameworks. When the main error source shifts between strong-dynamic state evolution and degraded-link observation conditions, a single model may suffer from response lag or insufficient covariance adjustment.

In contrast, IMM-RB updates mode probabilities with the assistance of link-state information and performs probabilistic fusion among representative estimation modes, such as normal, maneuvering, and degraded modes. In this way, residual-bias modeling, frequency-offset-assisted observation, and link-state adaptation are incorporated into the same recursive framework. Strong-dynamic intervals mainly correspond to rapid variations in propagation geometry, Doppler-related terms, and residual bias, whereas degraded-link intervals mainly correspond to reduced observation quality, changes in noise statistics, and local missing observations. Link-state scores provide interpretable inputs for mode-probability adjustment, allowing the estimator to maintain more stable error suppression and continuous output capability under different disturbance sources. Therefore, the advantage of IMM-RB over single-model filtering methods does not simply come from residual-bias state augmentation or filter-parameter tuning but mainly from the link-state-information-assisted multi-model adaptation mechanism. The role of frequency-offset-assisted observation is not merely to increase the number of observations but to improve the local numerical separability of residual-bias-augmented estimation. When only four-timestamp observations are used, the clock-offset state and the residual-bias state tend to enter the observation as a combined quantity, resulting in ill-conditioned directions in the local observation matrix. After frequency-offset-assisted observations are introduced, the minimum singular value and reciprocal condition number of the local observability matrix are improved, the correlation between the clock-offset state and the residual-bias state is reduced, and the clock-state uncertainty can maintain a finite bound under enhanced-noise conditions. This provides an observation-structure-level explanation for the improved synchronization accuracy and residual-bias tracking performance.

The current validation is mainly based on an equivalent stochastic-error simulation model implemented on a unified simulation platform. This setting enables the roles of different algorithmic mechanisms to be analyzed under controlled and reproducible conditions, but it is not a substitute for real-link or full physical-layer channel validation. In real microwave links, hardware-delay variations, receiver tracking errors, attitude disturbances, multipath effects, and non-Gaussian interference may introduce more complex error characteristics. Therefore, future work should further verify the engineering applicability of the proposed method using measured links or hardware-in-the-loop platforms, and calibrate the link-state scores, mode transition probabilities, and noise-inflation parameters using real link-state measurements. For stronger nonlinearity and multi-node cooperative scenarios, high-order nonlinear error propagation or data-driven residual modeling methods may be further incorporated [29,30], and the proposed framework can be extended to multi-link cooperative time synchronization systems.

## 6. Conclusions

This paper addresses the problem that post-compensation residual asymmetric errors in high-dynamic two-way microwave links are difficult to separate, model, and suppress. A residual-bias online estimation method jointly constrained by frequency-offset assistance and link-state information is proposed. The method transforms the post-compensation residual asymmetric error from an unmodeled disturbance into a recursively estimable dynamic state, and it introduces the residual-bias rate to characterize the dominant time-varying behavior within a short sampling interval. At the observation level, a four-timestamp and frequency-offset joint observation model is constructed, in which frequency-offset information is used as an observation-level auxiliary constraint to enhance the local separability among the clock offset, frequency offset, and residual link state. At the estimation level, a link-state-information-assisted IMM-IEKF is adopted. The maneuvering-intensity and link-degradation indicators are used to adjust the mode priors and noise covariances, enabling online joint estimation of clock parameters and link residual errors. The simulation results show that the proposed IMM-RB method can maintain low clock-offset estimation errors across multiple scenarios, exhibit more stable synchronization performance under composite disturbances and extreme high-dynamic conditions, and preserve continuous valid outputs under degraded-link conditions.

Overall, the proposed method provides simulation-based evidence for online modeling and suppression of post-compensation residual asymmetric errors in high-dynamic two-way microwave time synchronization. Its main contribution is to establish a unified framework centered on residual-asymmetry representation, observation-constraint enhancement, and link-state-information-assisted adaptive estimation. This framework enables residual link errors to be explicitly tracked and suppressed, providing a more interpretable online estimation scheme under high-dynamic and degraded-link simulation conditions.

## Figures and Tables

**Table 1:** Layered high-dynamic simulation scenarios and their engineering interpretation.

Scenario	Rel. Speed	Acc. *	Interpretation
E1-Nominal	300 m/s	2 m/s^2^	Low-to-medium high-dynamic microwave link
E2-HighDynamic	1500 m/s	10 m/s^2^	Rapid geometry variation and Doppler effects
E2-NoiseEnhanced	1500 m/s	10 m/s^2^	High-dynamic link with enhanced timestamp, path, and frequency-observation noise
E2-DegradedLink	1500 m/s	10 m/s^2^	High-dynamic link with local noise inflation and missing observations
E3-Composite	3000 m/s	20 m/s^2^	Strong dynamics with degraded observations
E4-Extreme	5000 m/s	35 m/s^2^	Upper-range applicability evaluation

* Rel. speed denotes the maximum equivalent relative speed of the microwave link.

**Table 2:** Main comparison methods.

Method	Description	Main Purpose
GC4TS	Geometric-compensated four-timestamp method	Conventional compensated
FA-noBias	Frequency-offset-aided method without residual-bias state	Tests the effect of frequency-offset assistance alone
Robust-IEKF-RB	Robust IEKF with residual-bias state	Single-model robust filtering baseline
VB-IEKF-RB	Variational-Bayes IEKF with residual-bias state	Single-model adaptive filtering baseline
IMM-RB	Link-state-information-assisted IMM-IEKF with residual-bias state	Proposed method

**Table 3:** Key parameters of the equivalent stochastic-error model and estimator.

Parameter	Setting
Baseline stochastic observation errors	$\varepsilon_{T}∼(0,\sigma_{T,0}^{2}),\varepsilon_{P}∼(0,\sigma_{P,0}^{2}),\varepsilon_{F,eq}∼(0,\sigma_{F,eq,0}^{2})$, $\sigma_{T,0}=40ps$, $\sigma_{P,0}=20ps$, $\sigma_{F,eq,0}=5ps$
Noise-enhanced and degraded-link settings	Noise-enhanced: $\sigma_{T,0}=120ps$, $\sigma_{P,0}=60ps$, $\sigma_{F,eq}=12ps$ Degraded link: $\left[t_{s},t_{e},\gamma_{T},\gamma_{F,eq},p_{miss}\right]$*, $\gamma_{T}=4-9,\gamma_{F,eq}=10-30,p_{miss}=0.15-0.78$
Residual-bias process	$b_{res,k}=a_{b}b_{res,k-1}+d_{k}+\sigma_{b}w_{k}$
Baseline Q/R setting	Q: mode-dependent diagonal covariance; $R_{0}=\mathrm{diag}(\sigma_{T}^{2}+\sigma_{P}^{2},0.5\sigma_{F}^{2},\sigma_{P}^{2})$
IMM and link-state adjustment	$\Pi_{ii}=0.955$, $\Pi_{ij}=0.0225,i\neq j$; $scoregain=2.5,\tau_{\deg}=0.50,\lambda_{\deg}=12.0$
Advanced baseline parameters	Robust-IEKF-RB: Huber C = 3.8, max inflation = 8 VB-IEKF-RB: forgetting factor = 0.996, scale range = 0.55^–10^

* $\sigma_{F,eq}$ denotes the equivalent time-domain standard deviation of the frequency-offset-assisted observation after residual-domain construction, rather than the raw frequency-offset measurement noise. $t_{s}$ and $t_{e}$ denote the start and end times of a degraded interval, $\gamma_{T}$ and $\gamma_{F,eq}$ denote the timestamp-noise and frequency-observation-noise amplification factors, and $p_{miss}$ denote the missing-observation probability. The degraded-link intervals are predefined by the scenario configuration rather than selected according to estimation results.

**Table 4:** Evaluation metrics used in the simulation.

Metric	Formula or Definition
Clock-offset error	$e_{\theta ,k}={\hat{\theta }}_{k}-\theta_{k}$
Overall clock-offset RMSE	$RMSE_{\theta }=\sqrt{\frac{1}{N}\sum_{k=1}^{N}e_{\theta ,k}^{2}}$
Window clock-offset RMSE	$RMSE_{\theta ,W}=\sqrt{\frac{1}{\left|W\right|}\sum_{k\in W}e_{\theta ,k}^{2}}$
Peak moving RMSE	$max\left(mRMSE_{\theta }\right)=\max_{k\in W}mRMSE_{\theta ,k}$
Peak absolute clock-offset error	${\left|e_{\theta }\right|}_{\max}=\max_{k\in W}\left|e_{\theta ,k}\right|$
Valid-output ratio	$r_{\mathrm{valid}}=\frac{N_{\mathrm{valid}}}{N_{W}}$
Residual-bias RMSE	$RMSE(b_{res})=\sqrt{\frac{1}{N}\sum_{k=1}^{N}e_{b,res,k}^{2}}$
Residual-bias MAE	$MAE(b_{res})=\frac{1}{N}\sum_{k=1}^{N}\left|e_{b,res,k}\right|$
Local numerical observability	$\sigma_{\min}(O),1/cond(O),\left|corr(\theta ,b_{res})\right|$
NIS consistency	$NIS_{k}=\nu_{k}^{T}S_{k}^{-1}\nu_{k}$
IEKF termination	$\varepsilon_{\mathrm{IEKF}}=10^{-3}$ $,I_{\max}=3$
Real-time factor	$\rho_{RT}=\frac{{\bar{t}}_{ep}}{\Delta t}$

**Table 5:** Local-window diagnostic statistics in the strong-dynamic interval.

Method	RMSE_θ,W_/ns	Max (mRMSE_θ_)/ns	Max|e_θ_|/ns	Valid Ratio
GC4TS	0.3498	0.5485	1.2455	1.0000
FA-noBias	0.3479	0.5667	1.3336	1.0000
Robust-IEKF-RB	0.2557	0.4738	0.4788	1.0000
VB-IEKF-RB	0.3149	0.5391	0.5413	1.0000
IMM-RB	0.2175	0.3819	0.3936	1.0000

**Table 6:** Local-window diagnostic statistics in the degraded-link interval.

Method	RMSE_θ,W_/ns	Max (mRMSE_θ_)/ns	Max|e_θ_|/ns	Valid Ratio
GC4TS	0.3955	0.7855	1.7509	0.7794
FA-noBias	0.3722	0.6312	1.7709	0.7794
Robust-IEKF-RB	0.1944	0.4158	0.4164	1.0000
VB-IEKF-RB	0.1852	0.5340	0.5056	1.0000
IMM-RB	0.1421	0.3166	0.3209	1.0000

**Table 7:** NIS-based innovation consistency of IMM-RB under Monte Carlo random seeds.

Scenario	Mean NIS	NIS 95% Coverage *
E2-HighDynamic	2.1625	0.9042
E2-DegradedLink	2.7967	0.8832
E3-Composite	3.5048	0.8829

* NIS denotes the normalized innovation squared. The NIS 95% coverage denotes the proportion of samples for which their NIS values fall within the theoretical 95% chi-square confidence interval. Under Gaussian noise with a fully calibrated innovation covariance, the expected coverage is 0.95; values lower than 0.95 indicate imperfect covariance calibration rather than direct filter divergence.

**Table 8:** Quantitative residual-bias estimation errors of IMM-RB across scenarios.

Scenario	RMSE(b_res_)/ps	MAE(b_res_)/ps	P_95_(|e_b,res_|)/ps	RMSE_deg_(b_res_)/ps
E2-HighDynamic	206.74	161.66	402.69	—
E2-DegradedLink	241.19	179.35	471.54	352.18
E3-Composite	296.25	219.82	619.43	414.25
E4-Extreme	342.49	254.60	733.81	449.90

**Table 9:** Computational cost and real-time feasibility of different methods.

Method	$n_{x}$	$N_{m}$	Cost Model	${\bar{t}}_{ep}$/ms	$\rho_{RT}$	$T_{rec}$/s
GC4TS	0	0	$C_{ep}\propto 1$	0.0004	$1.89\cdot 10^{-5}$	0.1050
FA-noBias	0	0	$C_{ep}\propto 1$	0.0001	$5.73\cdot 10^{-6}$	0.0950
Robust-IEKF-RB	5	1	$C_{ep}\propto In_{x}^{3}$ *	0.1590	$7.95\cdot 10^{-3}$	0.0200
VB-IEKF-RB	5	1	$C_{ep}\propto In_{x}^{3}$	0.1830	$9.18\cdot 10^{-3}$	0.0200
IMM-RB	5	3	$C_{ep}\propto N_{m}In_{x}^{3}+N_{m}^{2}n_{x}^{2}$	0.6420	$3.21\cdot 10^{-2}$	0.0200

* I denotes the number of IEKF iterations.

## Data Availability

The supporting data for this study can be obtained upon request from the corresponding author. Due to privacy concerns involving the participants, these data are not publicly available.
